# Supplementary material for: Inflammatory markers in world trade center workers with asthma: Associations with post traumatic stress disorder
Source: PLoS One. 2024 Feb 13;19(2):e0297616. doi: 10.1371/journal.pone.0297616 (PMC10863856; doi:10.1371/journal.pone.0297616)
Supplement: S1 Appendix — (DOCX) [file pone.0297616.s001.docx]

**Supporting Information**

**Table S1. Correlations Between Blood and Sputum Cytokine Levels and Post Traumatic Stress Disorder Checklist for DSM-5 Scores**

| **Cytokine** | **Blood** | | **Sputum** | |
| --- | --- | --- | --- | --- |
|  | **Correlation Coefficient** | **P-value** | **Correlation Coefficient** | **P-value** |
| IL-1α | -0.1 | 0.47 | 0.1 | 0.52 |
| IL-1β | 0.0 | 0.52 | 0.1 | 0.66 |
| IL-2 | 0.1 | 0.38 | 0.0 | 0.73 |
| IL-3 | 0.1 | 0.14 | 0.1 | 0.63 |
| IL-4 | 0.1 | 0.24 | 0.0 | 0.85 |
| IL-5 | 0.0 | 0.78 | -0.1 | 0.56 |
| IL-6 | 0.1 | 0.19 | 0.0 | 0.92 |
| IL-7 | 0.1 | 0.25 | 0.1 | 0.34 |
| IL-8 | 0.1 | 0.41 | -0.2 | 0.13 |
| IL-10 | 0.0 | 0.86 | 0.2 | 0.22 |
| IL-12 | 0.0 | 0.88 | 0.2 | 0.24 |
| IL12p70 | 0.0 | 0.68 | 0.1 | 0.25 |
| IL-13 | 0.0 | 0.75 | -0.2 | 0.16 |
| IL-15 | 0.0 | 0.95 | 0.2 | 0.12 |
| IL-17α | -0.1 | 0.50 | -0.1 | 0.48 |
| IL-1Rα | 0.1 | 0.33 | -0.1 | 0.63 |
| G-CSF | 0.1 | 0.20 | 0.1 | 0.41 |
| GM-CSF | 0.0 | 0.68 | 0.0 | 0.93 |
| IP10 | 0.0 | 0.93 | 0.3 | 0.02 |
| IFN-α2 | 0.1 | 0.41 | 0.1 | 0.52 |
| IFN–γ | 0.1 | 0.20 | 0.1 | 0.69 |
| Eotaxin | 0.1 | 0.16 | 0.1 | 0.23 |
| EGF | -0.1 | 0.47 | 0.0 | 0.73 |
| MCP1 | 0.1 | 0.32 | -0.1 | 0.64 |
| MIP1-α | -0.1 | 0.30 | 0.0 | 0.79 |
| MIP1-β | 0.0 | 0.95 | 0.0 | 0.89 |
| RANTES | -0.1 | 0.08 | 0.1 | 0.52 |
| TNF-α | 0.1 | 0.22 | 0.0 | 0.94 |
| TNF-β | 0.1 | 0.39 | 0.0 | 0.77 |
| VEGF | 0.0 | 0.55 | 0.1 | 0.58 |
| **Cell Count Differential** |  |  | **Correlation Coefficient** | **P-value** |
| Eosinophils (%) | - | - | 0.3 | 0.07 |
| Neutrophils (%) | - | - | 0.3 | 0.10 |
| Macrophages (%) | - | - | -0.2 | 0.19 |
| Lymphocytes (%) | - | - | 0.1 | 0.39 |

IL: interleukin, G-CSF: granulocyte colony stimulating factor, GM-CSF: granulocyte-macrophage colony-stimulating factor, IP: interferon gamma inducible protein, IFN: interferon, EGF: epidermal growth factor, MIP: macrophage inflammatory protein, TNF: tumor necrosis factor, VEGF: vascular endothelial growth factor

**Table S2. Adjusted Association between Blood and Sputum Cytokine Levels and Post Traumatic Stress Disorders Checklist for DSM-5 Scores**

| **Cytokine** | **Blood** | | **Sputum** | |
| --- | --- | --- | --- | --- |
|  | **Mean Difference** | **95% Confidence Interval** | **Mean Difference** | **95% Confidence Interval** |
| IL-1α | -1.0 | -5.4 to 3.3 | -1.1 | -5.3 to 3.0 |
| IL-1β | 0.03 | 0.01 to 0.06 | -0.4 | -1.0 to 0.2 |
| IL-2 | 0.03 | 0.01 to 0.06 | -0.01 | -0.03 to 0.01 |
| IL-3 | 0.01 | -0.001 to 0.01 | 0.002 | -0.01 to 0.01 |
| IL-4 | 3.5 | -6.2 to 13.3 | 0.1 | -0.2 to 0.3 |
| IL-5 | 0.01 | -0.1 to 0.1 | -0.02 | -0.1 to 0.02 |
| IL-6 | 0.03 | -0.7 to 0.7 | 0.02 | -0.7 to 0.7 |
| IL-7 | 0.1 | 0.02 to 0.1 | 0.2 | -0.1 to 0.5 |
| IL-8 | 0.1 | -0.2 to 0.4 | -19.3 | -66.2 to 27.6 |
| IL-10 | 0.1 | -0.1 to 0.3 | 0.1 | -0.01 to 0.2 |
| IL-12 | -5.1 | -14.5 to 4.3 | -0.1 | -0.3 to 0.1 |
| IL12p70 | -0.02 | -0.4 to 0.4 | -0.1 | -0.3 to 0.1 |
| IL-13 | -0.2 | -1.7 to 1.3 | -0.1 | -0.2 to 0.05 |
| IL-15 | 0.03 | -0.03 to 0.09 | -0.04 | -0.2 to 0.1 |
| IL-17α | 0.2 | -0.04 to 0.4 | -0.03 | -0.1 to 0.1 |
| IL-1Rα | 4.4 | -9.9 to 18.8 | -45.0 | -121.7 to 31.7 |
| G-CSF | 0.3 | -0.001 to 0.7 | 4.0 | -11.5 to 19.4 |
| GM-CSF | 0.01 | -0.1 to 0.1 | -0.0 | -0.1 to 0.03 |
| IP10 | -3.2 | -8.8 to 2.4 | 101.6 | -18.3 to 22.15 |
| IFN-α2 | 0.1 | -0.3 to 0.4 | 0.02 | -0.2 to 0.3 |
| IFN–γ | 0.3 | 0.05 to 0.6 | 0.1 | -0.02 to 0.1 |
| Eotaxin | 0.7 | -0.3 to 1.7 | 0.2 | -0.6 to 1.1 |
| EGF | -0.1 | -0.4 to 0.3 | -2.0 | -12.6 to 8.6 |
| MCP1 | 0.6 | -1.6 to 2.9 | 1.7 | -28.9 to 32.3 |
| MIP1-α | -0.1 | -0.2 to 0.1 | 0.2 | -1.6 to 2.0 |
| MIP1-β | -1.3 | -7.6 to 4.9 | 0.1 | -3.4 to 3.6 |
| RANTES | -24.6 | -51.2 to 2.0 | 0.1 | -0.1 to 0.3 |
| TNF-α | 0.1 | 0.02 to 0.2 | -0.02 | -0.3 to 0.3 |
| TNF-β | -0.2 | -3.0 to 2.5 | -0.01 | -0.1 to 0.1 |
| VEGF | 1.4 | 0.6 to 2.3 | 8.8 | -17.9 to 35.5 |
| **Cell Count Differential** |  |  | **Mean Difference** | **95% Confidence Interval** |
| Eosinophils | - | - | 0.1 | -0.2 to 0.4 |
| Neutrophils | - | - | -0.5 | -1.3 to 0.3 |
| Macrophages | - | - | 0.4 | -0.6 to 1.3 |
| Lymphocytes | - | - | 0.03 | -0.03 to 0.09 |

IL: interleukin, G-CSF: granulocyte colony stimulating factor, GM-CSF: granulocyte-macrophage colony-stimulating factor, IP: interferon gamma inducible protein, IFN: interferon, EGF: epidermal growth factor, MIP: macrophage inflammatory protein, TNF: tumor necrosis factor, VEGF: vascular endothelial growth factor
